# Supplementary material for: Spatiotemporal dynamics of lesion-induced axonal sprouting and its relation to functional architecture of the cerebellum
Source: Nat Commun. 2016 Sep 21;7:12938. doi: 10.1038/ncomms12938 (PMC5036008; doi:10.1038/ncomms12938)
Supplement: Supplementary Information — Supplementary Figures 1 - 4 [file ncomms12938-s1.pdf]

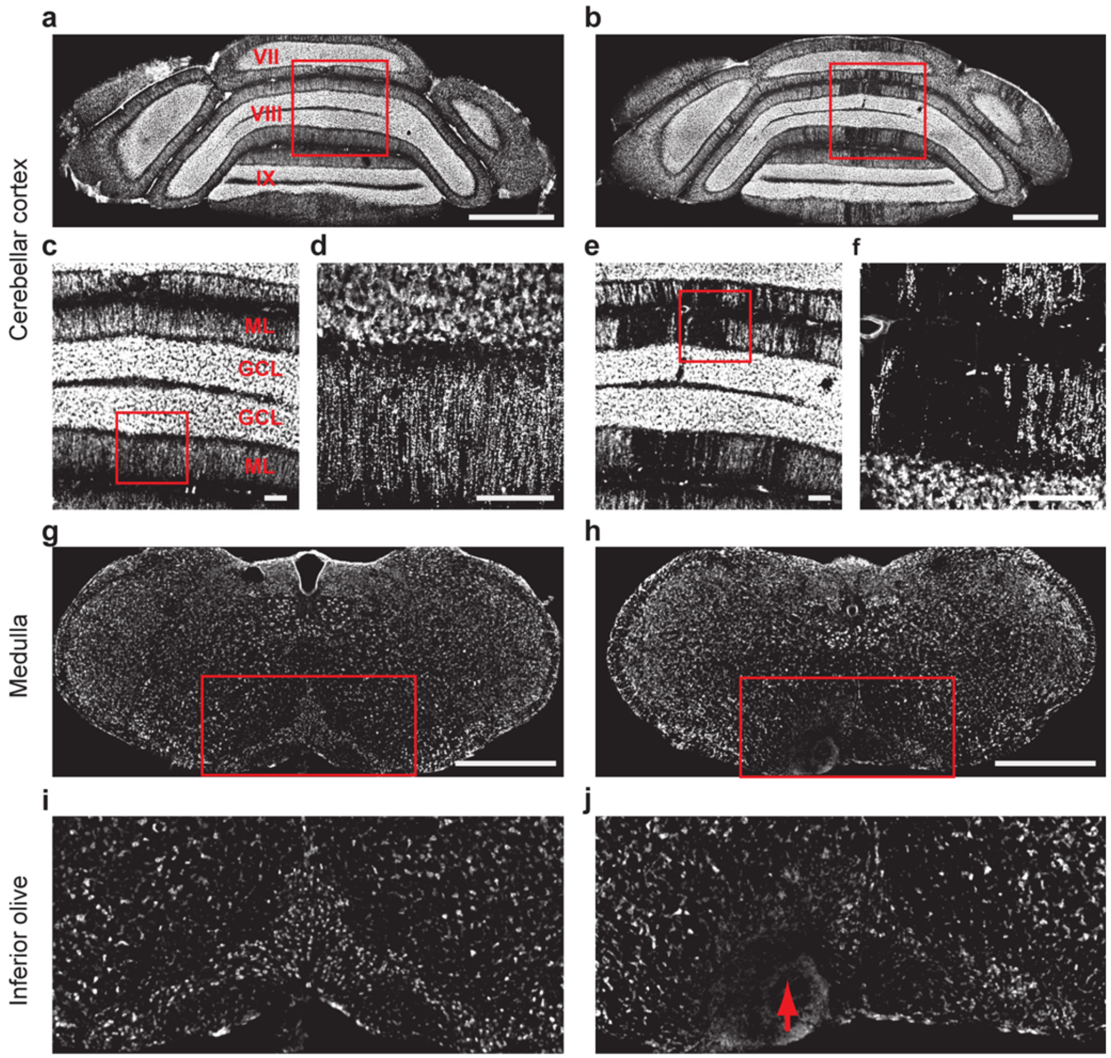

Supplementary Figure 1. CF lesion induced by 3-AP injection into the inferior olive. (**a-f**) Fixed coronal sections prepared from cerebellar cortex of naïve (**a, c, d**) and 3-AP injected (**b, e, f**) mice were immunostained with anti-VGLUT2 antibody to label all CF and mossy fiber terminals. The animal was sacrificed 1 week after 3-AP injection (n=2 animals). Sections containing lobule VII, VIII, and IX are shown with the vermal regions enclosed in the red squares in (**a**) and (**b**). These regions are magnified in (**c**) and (**e**), respectively. In (**c**), ML labels the molecular layer of the cerebellar cortex and GCL the granule cell layer. Corresponding layers of the cerebellar cortex are evident in (**e**). Regions enclosed in the red squares in (**c**) and (**e**) are further magnified in (**d**) and (**f**), respectively. While mossy fiber terminals in the granule cell layer were unaffected, a small population of CFs in the molecular layer degenerated. Scale bar for (**a, b**) is 1 mm and for (**c-f**) is 100 μm. (**g-j**) Fluorescent Nissl staining of coronal sections prepared from the medulla of naïve (**g, i**) and 3-AP injected (**h, j**) mice. The inferior olivary nucleus is enclosed in the red square in (**g, h**) and magnified in (**i, j**). The red arrow in (**j**) indicates the lesioned side of the nucleus. Although the 3-AP injection was unilateral, olivary neurons in the contralateral side are affected most likely because their axons cross the midline and pass through the lesioned area. Scale bar for (**g, h**) is 1 mm.

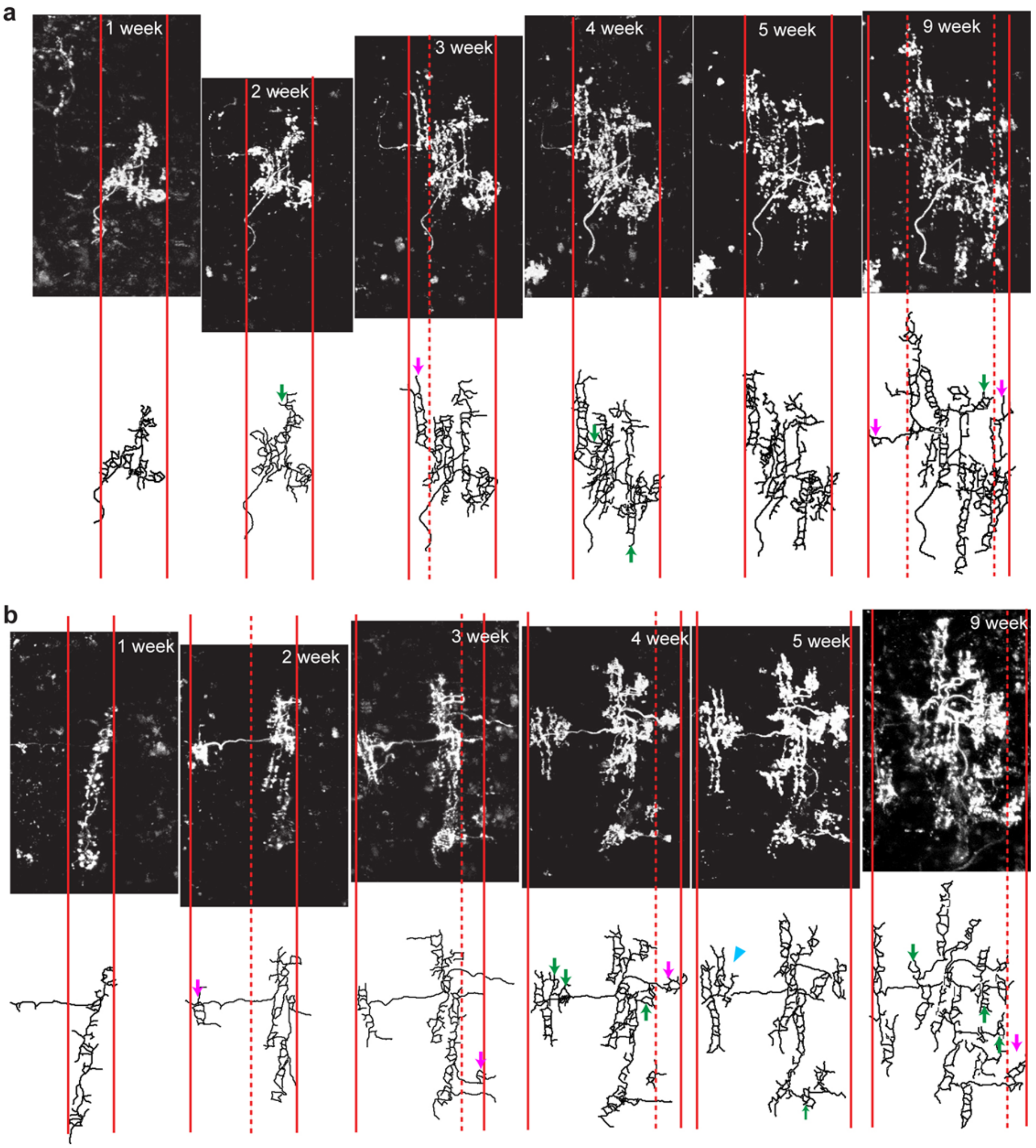

Supplementary Figure 2. Additional example traces for post-lesion CF collateral sprouting *in vivo*. (**a, b**) *In vivo* time-lapse images of the same surviving CFs and their traces for the time points mentioned at the top of the CF images. Maximum projections (top-down view) of the CFs in the molecular layer are shown. Solid red lines indicate the mediolateral extent of the CFs at each time point while the dashed red line indicates the mediolateral boundary from the previous time point that expanded in the current time point. Magenta arrows indicate ladders categorized as outside additions while green arrows indicate ladders categorized as inside additions. A blue arrowhead in (**b**) indicates an atypical CF ladder that later disappeared.

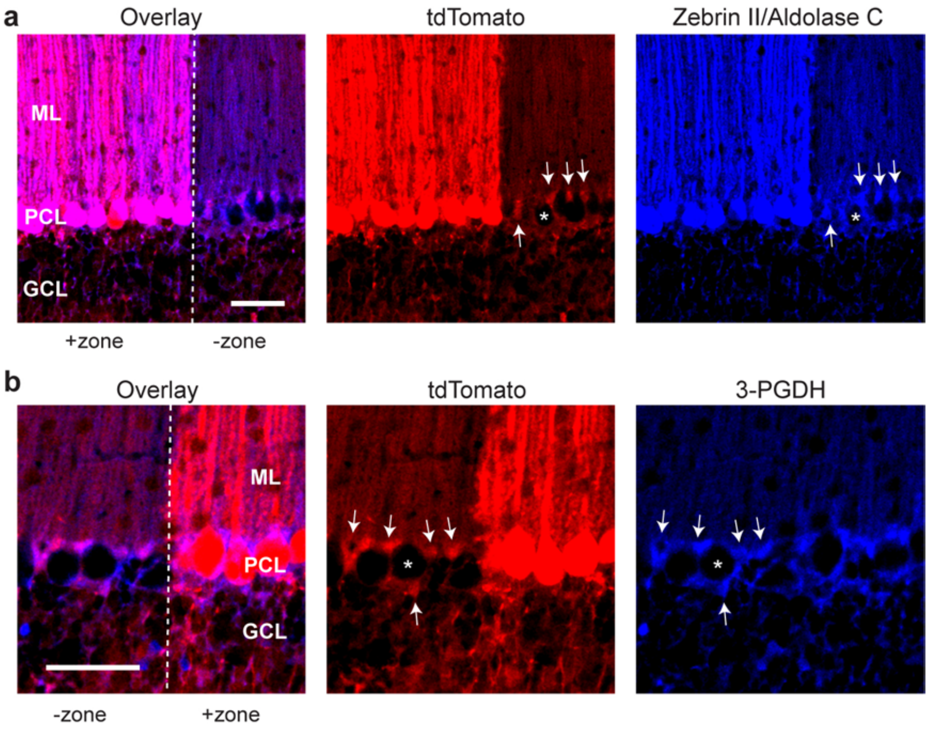

Supplementary Figure 3. Characterization of tdTomato expression in Aldoc-tdTomato × Nefl-EGFP double transgenic mouse. (**a, b**) Immunolabeling of zebrin II/aldolase C (blue) and 3PGDH (glial marker, blue) in lobule VIII of a fixed coronal section prepared from a double transgenic mouse. Since GFP fluorescence is observed in the double transgenic mouse, zebrin II/aldolase C and 3PGDH were both labeled with blue fluorescence in different sections to determine their overlap with the tdTomato (red) expression. (**a**) Red fluorescence was observed in tdTomato expressing PCs showing considerable overlap with zebrin II/aldolase C expressing PCs. The zonal boundary (white dashed line) identified by tdTomato expression matches with the boundary identified by zebrin II/Aldolase C expression. Small, Bergmann glia-like cells (white arrows) around tdTomato/zebrin II non-expressing PCs (asterisk) express both tdTomato and zebrin II, but the zonal boundary can be unambiguously identified nonetheless. (**b**) Small, tdTomato expressing cells (white arrows) around tdTomato/zebrin II non-expressing PCs (asterisk) express 3-PGDH, indicating that they are Bergmann glia. ML: molecular layer, PCL: Purkinje cell layer, GCL: granule cell layer. Scale bar is 50 μm.

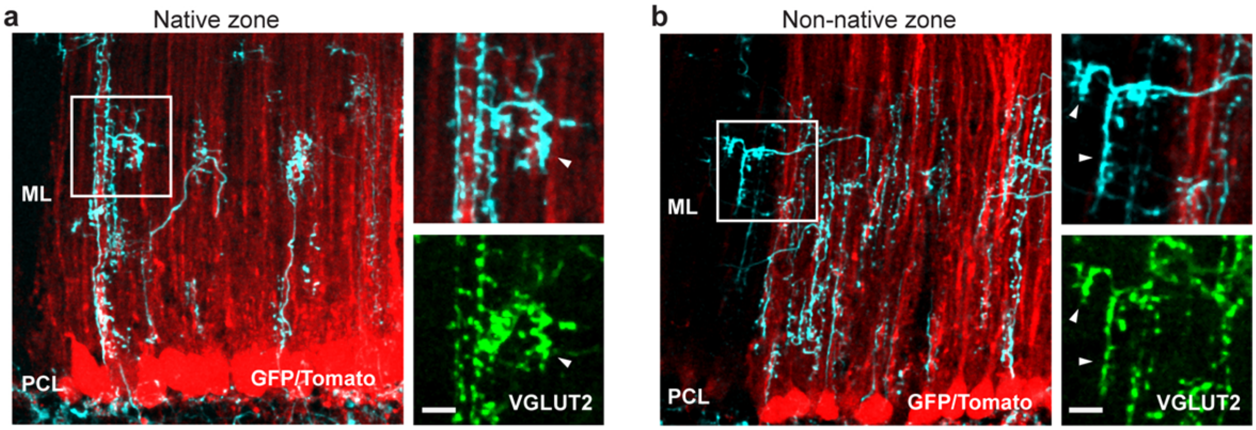

Supplementary Figure 4. New CF ladders in both native and non-native zones form synapses with PC dendrites. (**a, b**) Immunolabeling of synaptic sites with VGLUT2 (green) in an Aldoc-tdTomato × Nefl-EGFP double transgenic mouse sacrificed 2 weeks after 3-AP injection (n=2 animals). CFs (anti-GFP, cyan) and tdTomato-expressing PCs (red) in Lobule VIII are shown. (**a**) CF collateral sprouting within the native zone. The white square shows the site of a new CF ladder magnified on the right. Note that both the new CF ladder and the parent CF ladder form contacts with tdTomato expressing PCs and hence are within the native zone of the parent ladder. White arrowheads indicate the new CF ladder that expresses VGLUT2. (**b**) CF collateral sprouting in the non-native zone. The white square shows the site of new CF ladders magnified on the right. Note that the new CF ladders make contact with tdTomato non-expressing PCs in the non-native zone of the parent ladder. White arrowheads indicate the new CF ladders that express VGLUT2. ML: molecular layer, PCL: Purkinje cell layer. Scale bar is 10 µm.
